# Identification of key biomarkers based on the proliferation of secondary hyperparathyroidism by bioinformatics analysis and machine learning

**DOI:** 10.7717/peerj.15633

**Published:** 2023-07-10

**Authors:** Aiwen Shen, Jialin Shi, Yu Wang, Qian Zhang, Jing Chen

**Affiliations:** 1 Nephrology, Huashan Hospital, Fudan University, Shanghai, China; 2 National Clinical Research Center for Aging and Medicine, Huashan Hospital, Fudan University, Shanghai, China

**Keywords:** Secondary hyperparathyroidism, Machine learning, Immune cells infiltration, Biomarkers, Bioinformatics analysis

## Abstract

**Objective:**

Secondary hyperparathyroidism (SHPT) is a frequent complication of chronic kidney disease (CKD) associated with morbidity and mortality. This study aims to identify potential biomarkers that may be used to predict the progression of SHPT and to elucidate the molecular mechanisms of SHPT pathogenesis at the transcriptome level.

**Methods:**

We analyzed differentially expressed genes (DEGs) between diffuse and nodular parathyroid hyperplasia of SHPT patients from the GSE75886 dataset, and then verified DEG levels with the GSE83421 data file of primary hyperparathyroidism (PHPT) patients. Candidate gene sets were selected by machine learning screens of differential genes and immune cell infiltration was explored with the CIBERSORT algorithm. RcisTarget was used to predict transcription factors, and Cytoscape was used to construct a lncRNA-miRNA-mRNA network to identify possible molecular mechanisms. Immunohistochemistry (IHC) staining and quantitative real-time polymerase chain reaction (qRT-PCR) were used to verify the expression of screened genes in parathyroid tissues of SHPT patients and animal models.

**Results:**

A total of 614 DEGs in GSE75886 were obtained as candidate gene sets for further analysis. Five key genes (USP12, CIDEA, PCOLCE2, CAPZA1, and ACCN2) had significant expression differences between groups and were screened with the best ranking in the machine learning process. These genes were shown to be closely related to immune cell infiltration levels and play important roles in the immune microenvironment. Transcription factor ZBTB6 was identified as the master regulator, alongside multiple other transcription factors. Combined with qPCR and IHC assay of hyperplastic parathyroid tissues from SHPT patients and rats confirm differential expression of USP12, CIDEA, PCOLCE2, CAPZA1, and ACCN2, suggesting that they may play important roles in the proliferation and progression of SHPT.

**Conclusion:**

USP12, CIDEA, PCOLCE2, CAPZA1, and ACCN2 have great potential both as biomarkers and as therapeutic targets in the proliferation of SHPT. These findings suggest novel potential targets and future directions for SHPT research.

## Introduction

Secondary hyperparathyroidism (SHPT) is a frequent complication of chronic kidney disease (CKD) associated with morbidity and mortality (Cunningham, Locatelli & Rodriguez, 2011; Reiss et al., 2018). It starts in the early stages of CKD, and is characterized by the increased synthesis and secretion of parathyroid hormone (PTH) and the proliferation of gland cells (Naveh-Many & Volovelsky, 2020). SHPT has been shown to be associated with abnormal bone and mineral metabolism and cardiovascular disease (Block et al., 2004). By the time dialysis begins, most patients exhibit parathyroid hyperplasia and significantly increased PTH levels; these tended to increase with the extension of dialysis vintage (Tentori et al., 2015).

Parathyroid hyperplasia in uremia is a significant clinical challenge, leading to nodular hyperplasia and autonomous PTH secretion and subsequent increased morbidity and mortality (Egstrand et al., 2022). The pathogenesis of SHPT of CKD is multifactorial: hypocalcemia, phosphate retention, insufficient renal calcitriol production, and a disordered fibroblast growth factor 23–Klotho axis are the main factors that cause excessive growth of the parathyroid gland (PTG) (Centeno et al., 2019; Urakawa et al., 2006). Previous studies from our team suggest that cyclooxygenase 2-prostaglandin E2-EP2 (Cox2-PGE2-EP2) plays an important role in parathyroid hyperplasia (Zhang et al., 2019, 2011). Meanwhile, other signaling pathways such as mTOR and ERK/MAPK can also affect the synthesis and release of PTH leading to parathyroid hyperplasia in CKD (Chen et al., 2018; Volovelsky et al., 2016). The mechanism behind parathyroid cell proliferation in SHPT remains unclear, primarily due to the lack of an appropriate cell culture model to study the proliferation and apoptosis of parathyroid cells (Drüeke, 2000).

In the early stages of SHPT, the parathyroid gland shows diffuse and polyclonal hyperplasia. However, long-term uremic stimulation promotes the proliferation of parathyroid cells from diffuse growth to nodular growth (Hassan et al., 2022). Previous studies have reported that nodular hyperplasia progresses faster and has more significant proliferative activity than purely diffuse hyperplasia (Ohta et al., 1994; Yamaguchi, Yachiku & Morikawa, 1997). Therefore, it is crucial to search for biomarkers to delay the progression of proliferative parathyroid glands from diffuse to nodular. Meanwhile, there is currently no comprehensive biological information research on the SHPT. The development of bioinformatics analysis and machine learning makes it possible to explore the potential mechanism of diseases on a large scale at the transcriptome level.

In this study, we aimed to determine the detailed molecular mechanism of SHPT-related genes. With microarray data obtained from the Gene Expression Omnibus (GEO) database, we first applied machine learning to identify key genes between diffuse and nodular hyperplasia tissues. Immune cell infiltration and transcription factors analysis of key genes allowed further investigation of the molecular mechanism of parathyroid gland proliferation. Correlation between key genes and parathyroid-related disease-causing genes verified that these key genes were significantly correlated to parathyroid diseases. Next, the predictive performance of key genes was performed to predict the occurrence and development of SHPT. Finally, we conducted corresponding experiments to verify the expression of these five key genes in parathyroid tissues of SHPT patients and animal models.

## Materials and Methods

### Human parathyroid tissue preparations

Parathyroid tissues were obtained between 2019 and 2022 from ten hemodialysis patients diagnosed with SHPT who underwent parathyroidectomy surgery, following approval from the Ethics Committee on Human Research of the Huashan Hospital, Fudan University, China (KY2018-369). Ultrasonography confirmed the presence of one or more PTGs >1 cm^3^. All participants signed an informed consent form after understanding their rights, the risks when participating in this study, and the purpose and process of our research. Immediately after resection, a piece of parathyroid tissue was taken from each gland, fixed with 4% paraformaldehyde, and embedded in paraffin for subsequent histological study. The rest tissues were frozen and stored at −80 °C. Each sample contains only one parathyroid tissue from one patient in subsequent immunohistochemistry and qRT-PCR testing. Basic demographics are shown in Table S1.

### Animal model

All animal experiments were approved by the Ethics Committee for Animal Care and Use of Fudan University (No. 20171300A243). Male Sprague–Dawley rats (200–250 g) were purchased from Vital River Laboratory Animal Technology Co. Ltd. (Beijing, China). All the animals were housed in an environment with a temperature of 22 ± 1 °C, relative humidity of 50 ± 1%, and a light/dark cycle of 12/12 h. We randomly divided the animals into two groups of five animals each. A 5/6-nephrectomy (Nx) rat fed with high dietary phosphate is a commonly used animal model of human SHPT (Zhang et al., 2011). A 5/6-Nx was performed in a two-step procedure (Shvil et al., 1990). Following the second surgery, five rats were given a high-phosphate (1.2%) diet for 4 weeks. A control group consisted of five sham-operated rats fed with normal-phosphate diets (0.8%). Both the normal and high phosphate diets were obtained from Fanbo Biotechnology Co. (Wuxi, China) and contained the same levels of calcium (1.0%), vitamin D (750 IU/kg), lipids, and other nutrients. After a 4 weeks observation period, rats were anesthetized and sacrificed with sodium pentobarbital through intraperitoneal infection, and blood samples were obtained for creatinine, blood urea nitrogen (BUN), and intact PTH (iPTH). The PTGs were immediately removed by microdissection equipment. All rats were sampled in 1 day.

### Data download

The GEO series matrix file GSE75886 was downloaded from the NCBI GEO public database, and the annotation file GPL10558. Thirteen patients of samples were included in the expression profile data; these consisted of five cases of hemodialysis patients with diffuse parathyroid hyperplasia, and eight cases of hemodialysis patients with nodular hyperplasia. We also downloaded the series matrix file GSE83421 and the annotation file GPL22020 for verification of key gene expression levels. In total, groups of samples were included in the expression profile analysis: six normal groups and 25 parathyroid adenoma patients.

### Screening of key genes

The key genes identified from the GEO dataset were screened by differential analysis (*p* value < 0.05 and |logFC| >1), and the candidate gene sets were further screened by random forest (randomForest) and support vector machine (SVM) algorithms. Random forest is an ensemble learning algorithm with a decision tree as the base learner. It selects multiple samples from the sample set as a training set using the method of sampling and replacement, and uses the sample set obtained by sampling to generate a decision tree. At each generated node, it selects features randomly and without repetition, and then uses these features to divide the sample set, find the best dividing feature, and determine the predicted result. In this study, a random forest algorithm was used to evaluate the importance of candidate gene sets. A total of 1,000 classification trees were constructed, stirred 50 times, and evaluated according to the percentage of the increased mean-squared error (%IncMSE). SVM-Recursive Feature Elimination (SVM-RFE) is a machine learning method based on support vector regression analysis. SVM-RFE identifies the best variable (biomarker) by recursively removing variables to establish a more strongly supported classification model through the “e1071” software package which then further identifies the effect of these biomarkers on the disease. Following RF and SVM-RFE we kept the top-ranked features for subsequent analysis.

### Functional annotation of differential gene sets

DEGs were annotated using the Metascape database (www.metascape.org). This allows a thorough exploration of the functional relevance of genes. Gene Ontology (GO) analysis and Kyoto Encyclopedia of Genomes (KEGG) pathway analysis were also performed. A min overlaps greater than three and & pgreat were considered statistically significant. Min Overlap refers to the minimum allowable number or proportion of overlap between gene sets when comparing different gene lists. That is, if the number or proportion of overlap between two gene sets is lower than the set minimum threshold, it is not considered meaningful enrichment. Gene set enrichment analysis (GSEA) works based on a predefined set of genes. It sorts genes based on their differential expression between two groups, and then assesses whether the predefined gene set is more heavily associated with the top or bottom of the ranking list. In this study, GSEA was used to compare the differences in signaling pathways identified with KEGG and GO between the diffuse and nodular hyperplasia patients, and to explore the molecular mechanism of core genes between the two groups. The number of substitutions was set to 1,000, and the substitution type was set to phenotype.

### Analysis of immune cell infiltration

The relative proportions of 22 types of immune infiltrating cells in patients in different subgroups were inferred through the analysis of gene expression data using the CIBERSORT algorithm. Pearson correlation analysis was performed on the risk score and immune cell content.

### Regulatory network analysis of key genes

The R package “RcisTarget” was used to predict transcription factors. Calculations with RcisTarget are based on motifs, and the normalized enrichment score (NES) for these motifs depends on the total number annotated in the database. These motifs may be annotated either from the source annotated data file, or based on gene sequences similar to annotated motifs. To assess the overexpression of a motif in a dataset, the area under the curve (AUC) for each motif-motif set pair was calculated from the recovery curves of the gene sets for motif ordering. The NES for each motif was then calculated based on the AUC distribution of all motifs in the dataset. rcistarget.hg19.motifdb.cisbpont.500bp from the gene-motif rankings database was used.

### Network prediction of lncRNA-miRNA-mRNA

Predict microRNA (miRNA) interaction pairs through the miRWalk database. In addition, in conjunction with TargetScan and miRDB databases, miRNAs shared by the three databases were selected as target miRNAs for key genes. Then, lncRNA was predicted through the ENCORI database, combined with lncRNA miRNA interactions and mRNA miRNA interactions to establish a lncRNA miRNA mRNA network, and visualized using Cytoscape.

### Histologic studies

Part of the PTG tissues was embedded in paraffin and cut into 3 µm sections for hematoxylin and eosin (H&E) and immunohistochemistry (IHC) staining. The sections were stained using standard H&E and IHC protocols as previously described (Kan et al., 2018). According to the characteristics of HE staining, the parathyroid glands of SHPT patients were divided into diffuse and nodular hyperplasia (Mao et al., 2021). The primary antibodies used were CIDEA (1:100; Affinity, West Bridgford, UK), CAPZA1(1:50, Proteintech, Manchester, UK), and PCOLCE2 (1:100; Wuhan Fine Biotech, Hubei, China).

### Quantitative real-time polymerase chain reaction (qRT-PCR)

Total RNA was isolated both from patient and rat parathyroid tissues using Trizol (Invitrogen) according to the manufacturer’s protocols and reverse transcribed with PrimeScript™ RT Master Mix (Takara). qRT-PCR was carried out with TB Green Premix Ex Taq (Takara). Primer sequences are given in Tables S2 and S3.

### Laboratory measurements

Serum creatinine and blood urea nitrogen (BUN) levels were determined using an autoanalyzer (Siemens). Intact PTH (iPTH) was measured with rat-specific enzyme-linked immunosorbent assay (ELISA) kits (Immutopics).

### Statistical analysis

All statistical analyses were performed in R studio (version 3.6). All results are expressed as mean ± SD. All statistical tests were two-sided and were performed using a *t*-test to determine *p*-values. Graphs were generated using GraphPad Prism 9. *p* < 0.05 was considered statistically significant.

## Results

### Identification of key genes

**Figure 1:**
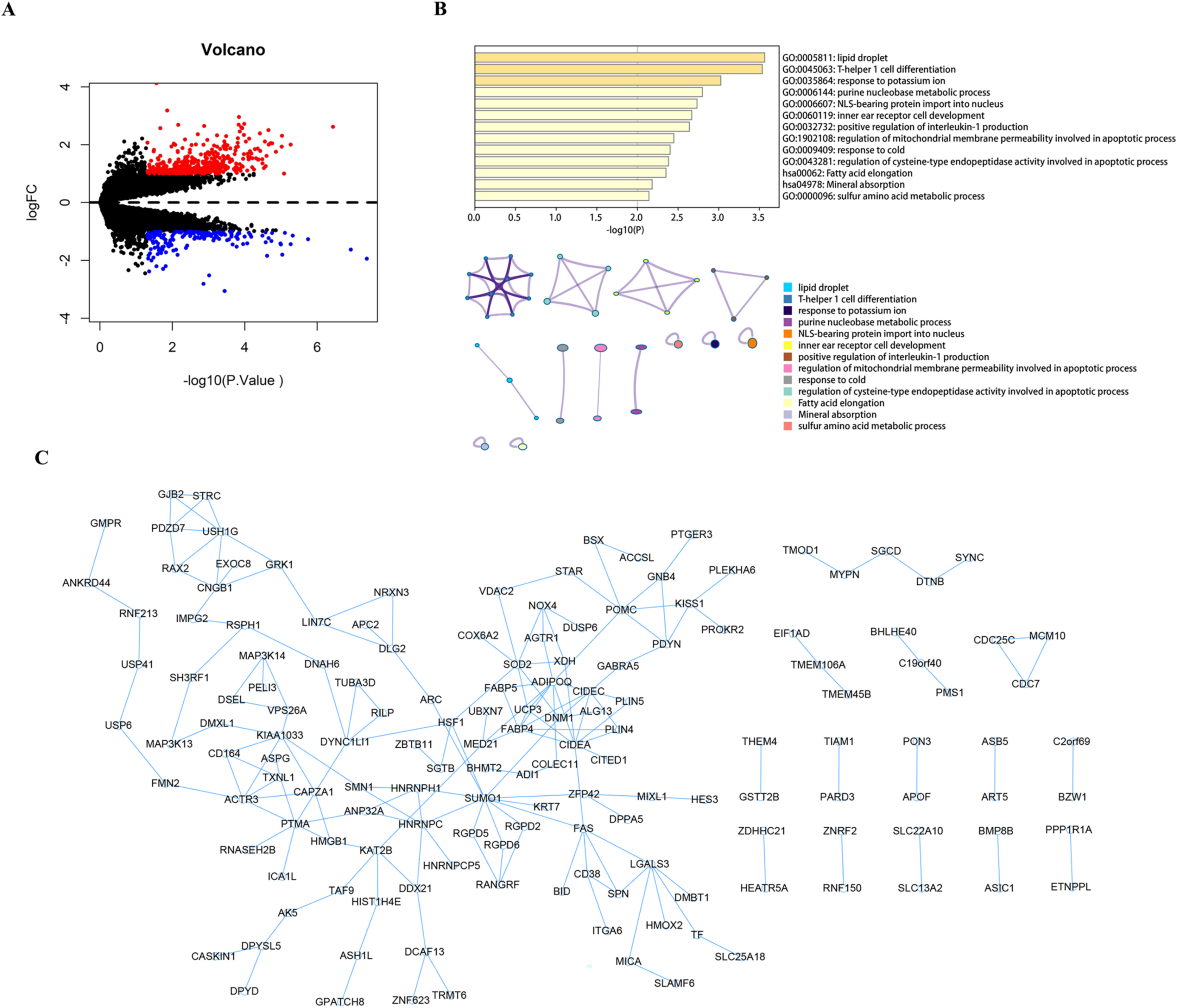
Analysis and function enrichment of DEGs. (A) Volcano plot identifies DEGs. Red dots represent upregulated genes and blue dots represent downregulated genes from nodular hyperplasia compared with diffuse hyperplasia parathyroid glands in SHPT patients. (B) Pathways anlalysis of DEGs was constructed using the Metascape database. Small networks and colorful names are cluster networks of enrichment paths. (C) The PPI network of DEGs was constructed using Cytoscape. Nodes represent proteins encoded by differential genes, and edges represent interactions between proteins. DEG, differentially expressed gene.

Using the limma package to count the differential genes between the hyperparathyroidism nodular hyperplasia and diffuse hyperplasia samples in the GEO dataset GSE75886, with differential gene screening conditions *p* value < 0.05 and |logFC| >1, a total of 428 up-regulated genes and 186 down-regulated genes were screened out (Fig. 1A). These 614 differential genes were used as candidate genes for further analysis. Pathway analysis of candidate genes was performed through the Metascape database. The results showed that these candidate genes were enriched in lipid droplets, T-helper one cell differentiation, response to potassium ions, and other pathways (Fig. 1B). We then performed protein interaction network analysis on the genes in the candidate gene set with Cytoscape software (Fig. 1C).

**Figure 2:**
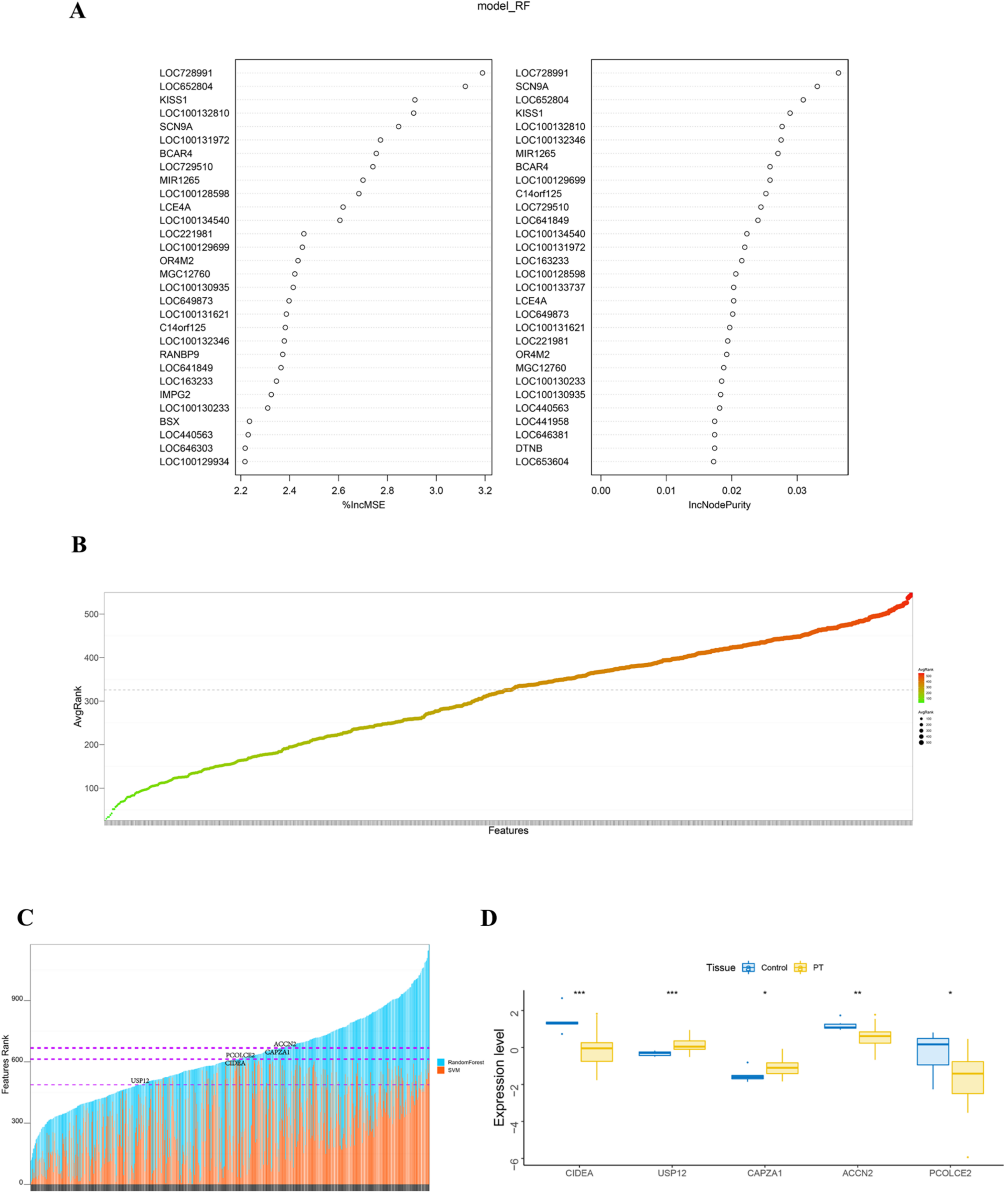
Identification and validation of key genes. (A) RandomForest analysis on DEGs. The left panel shows %IncMSE of random forest, and the right shows IncNodePurity. %IncMSE represents the percentage increase in the mean square error (MSE) of the model after removing each variable, which is the importance of each variable. IncNodePurity represents the increase in impurity of each node splitting after deleting each variable, which is the importance of each variable. A higher %IncMSE or IncNodePurity indicates that the variable contributes more to the model. model_RF: model random forest. (B) SVM analysis on DEGs. The bubble chart of DEGs ranking in SVM algorithm. The horizontal axis represents the gene name, and the vertical axis represents the gene ranking. (C) The sum of features rank from randomForest and SVM analyses. The horizontal axis represents DEGs, and the vertical axis represents the total ranking of DEGs. (D) The key genes with significant expression and the best ranking in the machine learning process were screened in the validation set GSE83421. *p < 0.05, **p < 0.01, ***p < 0.001.

The randomForest and SVM analysis further screened the 614 differential genes based on relation to disease disturbance (Figs. 2A and 2B). It was found that the sum of the ranking values obtained from the two analyses was distributed dispersedly (Fig. 2C). To more accurately screen genes related to the molecular mechanism of parathyroid glands, we performed a between-group rank-sum (Wilcox) test on the expression levels of the 614 differential genes in the validation set GSE83421; this dataset included six normal parathyroid glands and 25 adenomas from primary hyperparathyroidism (PHPT) patients. The key genes with a significant expression between groups and the best ranking in the machine learning process were identified as USP12, CIDEA, PCOLCE2, CAPZA1, and ACCN2 (Fig. 2D).

### Analysis of key genes with immune infiltration

**Figure 3:**
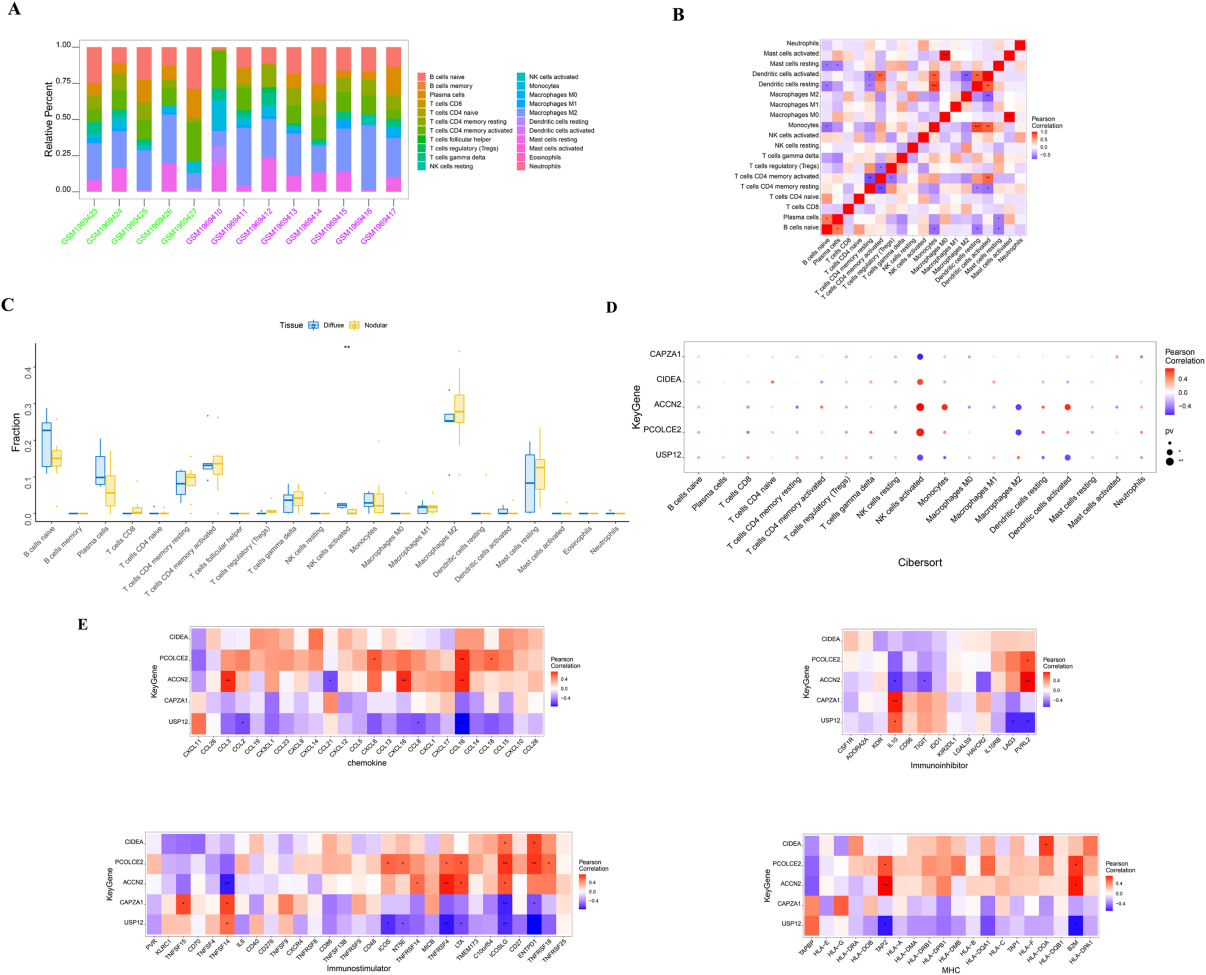
Immune cell infiltration in nodular and diffuse hyperplasia tissues, and immune association analysis of key genes. (A) The composition of 22 kinds of immune cells in each sample was showed in a histogram. (B) The correlation of 22 types of immune cells in parathyroid tissues was evaluated. Red: positive correlation; blue: negative correlateion. (C) Immune cell comparison between nodular and diffuse hyperplasia tissues. (D) Immune cell infiltration of key genes. (E) The correlations between key genes and different immune factors, including chemokines, immunoinhibitors, immunostimulators and major histocompatibility complex (MHC). *p < 0.05, **p < 0.01, ***p < 0.001.

SHPT causes an increase in PTH release and the presence of PTH receptors on lymphocytes, leading to impaired B cell proliferation in patients with chronic kidney failure (Alexiewicz et al., 1990). There is an interactive relationship between SHPT and immune cells (Alexiewicz et al., 1990; Cozzolino et al., 2008; Komaba, Kakuta & Fukagawa, 2017), and when treating SHPT, it is necessary to pay attention to changes in immune cells to maintain the normal function of the immune system. The molecular mechanisms of the key genes affecting parathyroid progression were explored by looking at the relationship between these genes and immune infiltration in the parathyroid dataset. The proportion of immune cells in each patient was shown in Figs. 3A and 3B. When compared with the patients with diffuse parathyroid hyperplasia, the level of natural killer cells (NK) activated was significantly lower in the nodular hyperplasia group (Fig. 3C).

We further identified several key genes that were highly correlated with immune cells: CIDEA, PCOLCE2, and ACCN2 were all positively correlated with NK cell activation; USP12 and CAPZA1 showed a negative correlation with NK cell activation; and PCOLCE2 and ACCN2 were negatively correlated with macrophages M2 (Fig. 3D). We further observed correlations between these genes and different immune factors from the TISIDB database such as immunomodulators and chemokines (Fig. 3E). These analyses further support the close relationship between the identified key genes and immune cell infiltration levels and suggest that they may play important roles in the immune microenvironment.

### GSEA analysis of key genes

**Figure 4:**
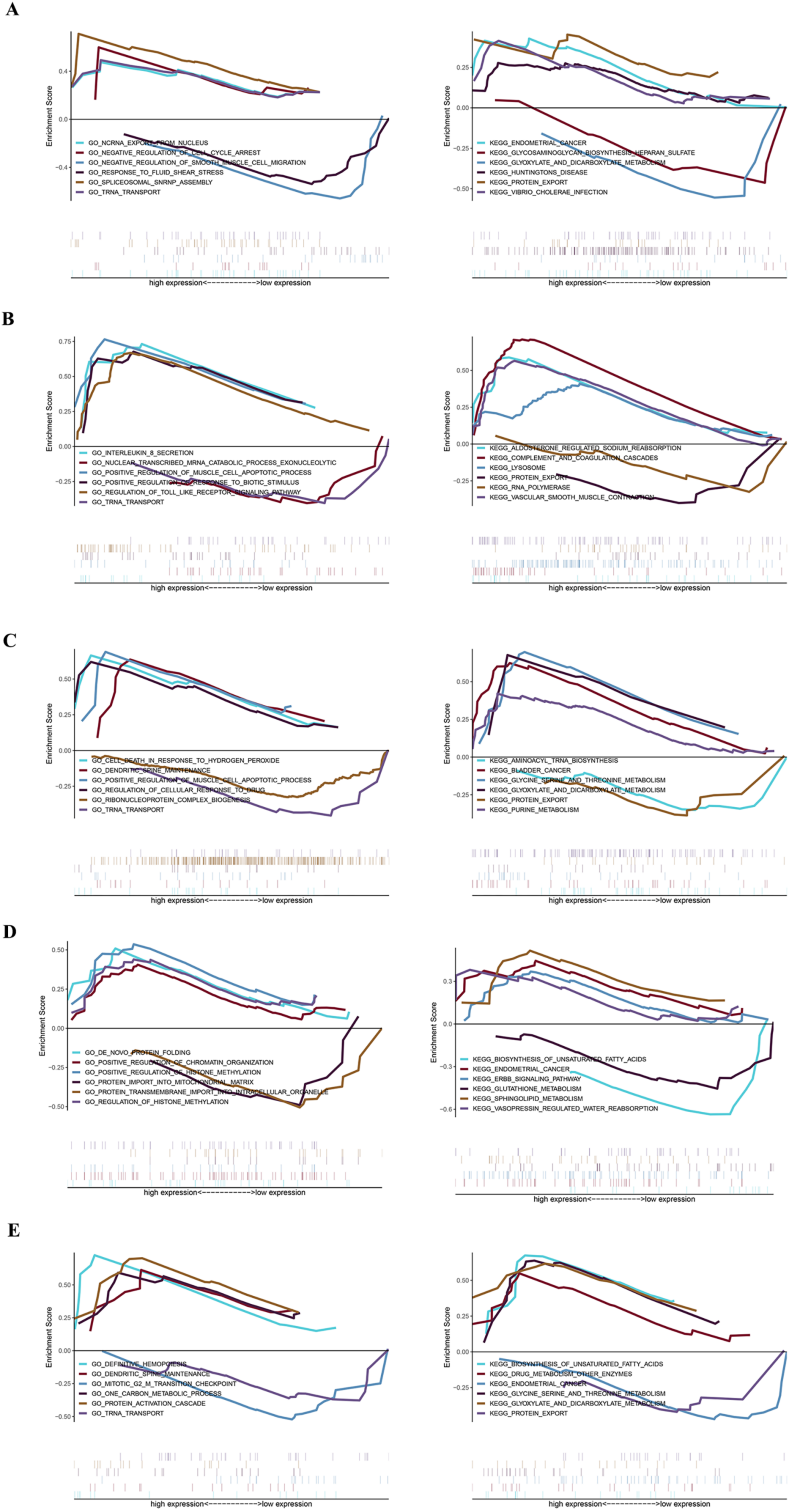
GSEA plot showing most enriched GO terms and KEGG pathways of key genes. The GSEA results of (A) USP12, (B) CIDEA, (C) PCOLCE2, (D) CAPZA1 and (E) ACCN2.

The specific signaling pathways enriched by the five identified key genes and the potential molecular mechanisms of core genes affecting parathyroid progression were further explored with GSEA analysis. The USP12 gene GO-enriched pathways included “ncRNA export from nucleus” and “negative regulation of cell cycle arrest,” and the KEGG-enriched pathways included “endometrial cancer” and “glycosaminoglycan biosynthesis heparan sulfate,” among others (Fig. 4A). The CIDEA gene GO-enriched pathways included “interleukin-8 secretion” and “nuclear-transcribed mRNA catabolic process exonucleolytic,” and the KEGG-enriched pathways includes “aldosterone regulated sodium reabsorption” and “complement and coagulation cascades” (Fig. 4B). The PCOLCE2 gene GO-enriched pathways included “cell death in response to hydrogen peroxide” and “dendritic spine maintenance,” and the KEGG-enriched pathways included “aminoacyl tRNA biosynthesis” and “bladder cancer” (Fig. 4C). The GO-enriched pathways of the CAPZA1 gene included “*de novo* protein folding” and “positive regulation of chromatin organization,” and the KEGG-enriched pathways included “biosynthesis of unsaturated fatty acids” and “endometrial cancer” (Fig. 4D). The ACCN2 gene GO-enriched pathways included “definitive hemopoiesis” and “dendritic spine maintenance,” and the KEGG-enriched pathways included “biosynthesis of unsaturated fatty acids” and “drug metabolism other enzymes” (Fig. 4E).

### Enriched motifs and corresponding transcription factors for key genes

**Figure 5:**
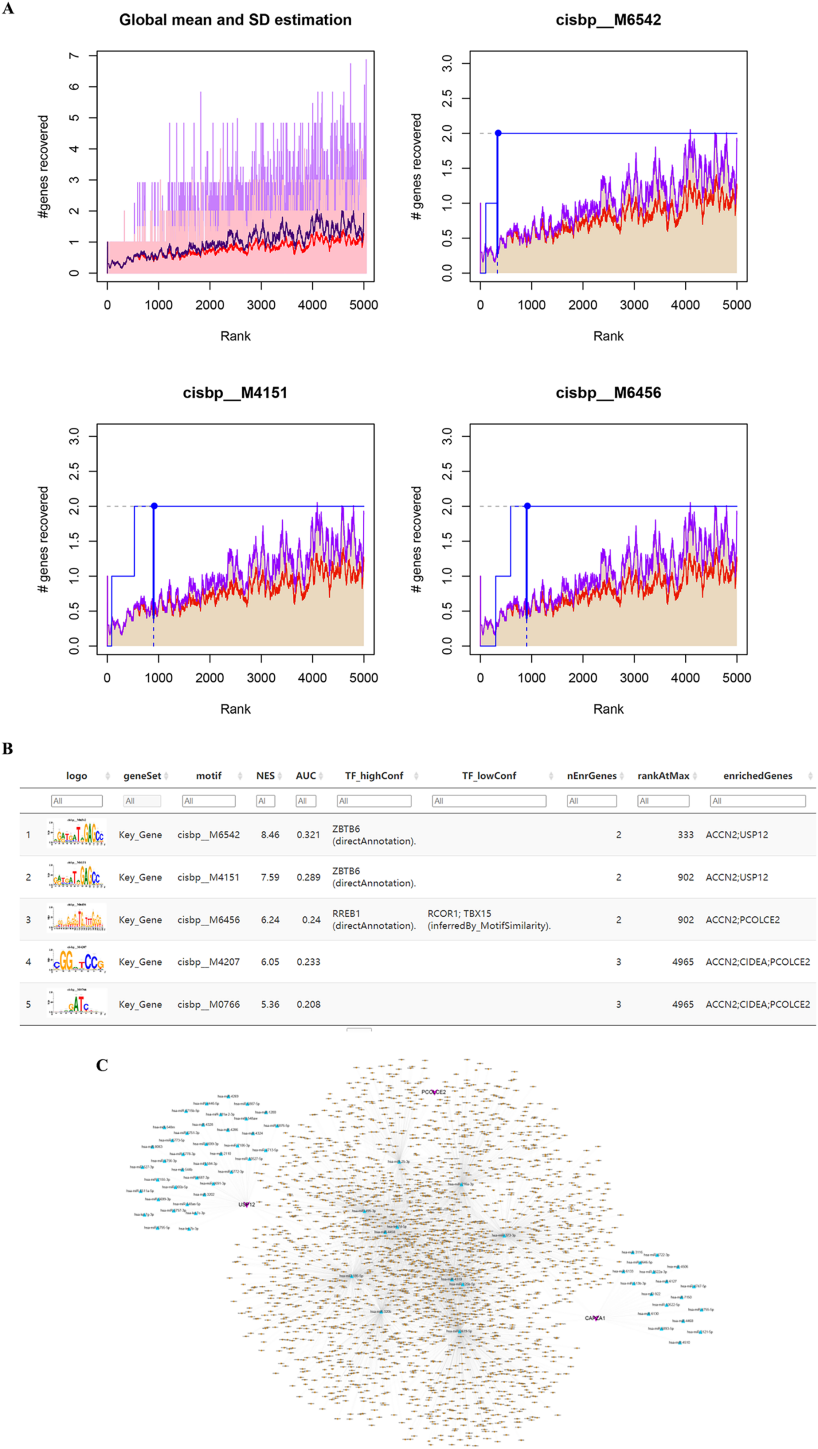
Motif prediction and miRNAs-lncRNAs of key genes. (A) The transcription factors were enriched using cumulative recovery curves. (B) The top five enriched motifs and corresponding transcription factors of NES for key genes. (C) The possible miRNAs and lncRNAs prediction and analysis of five key genes through miRWalk database and ENCORI database respectively, and a ceRNA network was constructed by cytoscape.

The five identified key genes were found to be regulated by multiple common transcription factors which were identified by searching for enriched motifs using cumulative recovery curves and selection of important genes (Fig. 5A). The analysis showed that the motif with the highest normalized enrichment score (NES: 8.46) was annotated as cisbp_M6542, and the transcription factor ZBTB6 was identified as the master regulator. Two of the five genes were enriched in this motif: ACCN2 and USP12. All enriched motifs and corresponding transcription factors for the key genes are shown in Fig. 5B.

### miRNAs and lncRNAs prediction of key genes

The prediction and analysis of the miRWalk database and ENCORI database were used to obtain the possible miRNAs and lncRNAs of these five key genes, respectively. First, the mRNA-related mRNA-miRNA relationship pairs were extracted from the miRWalk database, giving a total of 993 mRNA-miRNA relationship pairs of which 68 relationship pairs were retained (including three mRNA relationship pairs that were verified in TargetScan or miRDB and 68 miRNAs). Based on these 68 miRNAs, the interacting lncRNAs were predicted, resulting in a total of 4,152 predicted pairs of interactions (including 11 miRNAs and 2,224 lncRNAs). Finally, we constructed a ceRNA network in Cytoscape v3.7 (Fig. 5C).

### Correlation between key gene and parathyroid-related disease-causing genes

**Figure 6:**
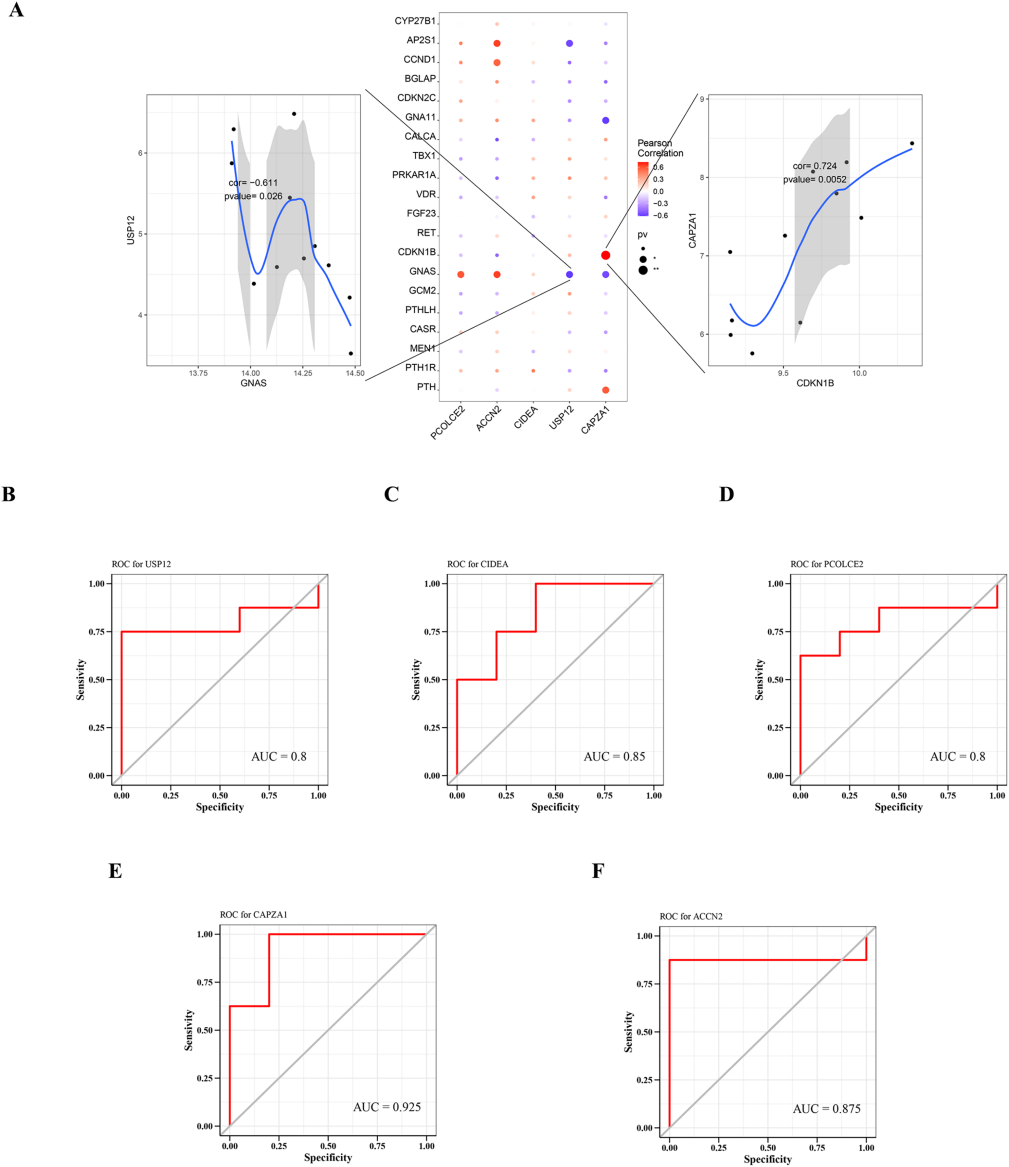
Relationship between key genes and parathyroid-related disease-causing genes, and predictive performance of key genes. (A) Correlation between key gene and parathyroid-related disease-causing genes. ROC analysis of (B) USP12, (C) CIDEA, (D) PCOLCE2, (E) CAPZA1 and (F) ACCN2.

A total of 2,333 parathyroid-related disease-causing genes were obtained from the GeneCards database (https://www.genecards.org/), and selected the top 20 genes with relevant scores to have a further analysis with the expression levels of the five key genes. The gene expression levels of key genes were found to be significantly correlated with the expression levels of multiple disease-related genes. Among them, CAPZA1 was positively correlated with CDKN1B (cor = 0.724), and USP12 was negatively correlated with GNAS (cor = −0.611) (Fig. 6A).

### Predictive performance of key genes

The predictive performance of key genes was explored using the ROC curves of diagnostic efficacy validation; higher AUC values suggest better predictive performance. The AUC values of the five key genes were: USP12 = 0.8 (Fig. 6B), CIDEA = 0.85 (Fig. 6C), PCOLCE2 = 0.8 (Fig. 6D), CAPZA1 = 0.925 (Fig. 6E), and ACCN2 = 0.875 (Fig. 6F). These high AUC values suggest that these five key genes may be good predictors of the occurrence and development of disease.

### Validation of key genes in different types of hyperplastic parathyroid tissues from uremic patients

**Figure 7:**
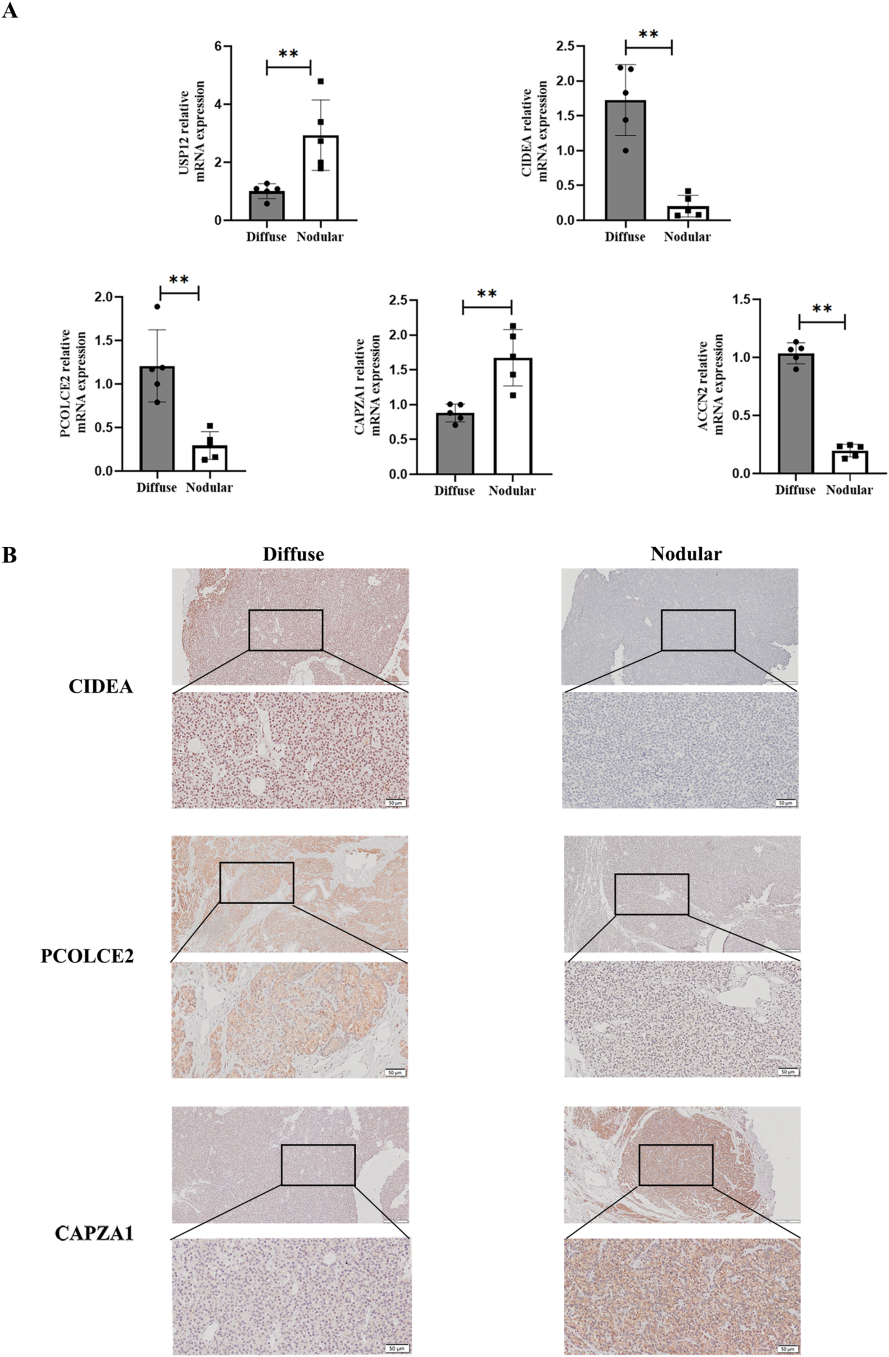
Validation of key gene in parathyroid tissues from SHPT patients. (A) USP12, CIDEA, PCOLCE2, CAPZA1 and ACCN2 as examined by qRT-PCR assay. (B) IHC staining to validate expression levels of CIDEA, PCOLCE2 and CAPZA1. *n* = 5, **p < 0.01.

To determine the expression of key genes in parathyroid glands in SHPT, qPCR was used to perform differential analysis between diffuse and nodular hyperplasia tissues. The results showed that gene expression of the five key genes (USP12, CIDEA, PCOLCE2, CAPZA1, and ACCN2) differed between diffuse and nodular hyperplasia tissues (Fig. 7A); CIDEA, PCOLCE2, and ACCN2 gene expression was significantly higher in diffuse hyperplasia tissues than in nodular hyperplasia samples. We further examined the expression of CIDEA, PCOLCE2, and CAPZA1 by IHC (Fig. 7B) and found that CAPZA1 was up-regulated in nodular hyperplasia of uremia patients, showing a more brownish patchy or granular staining when compared to that in the diffuse hyperplasia group.

### Key Genes expression in animal models of SHPT

**Figure 8:**
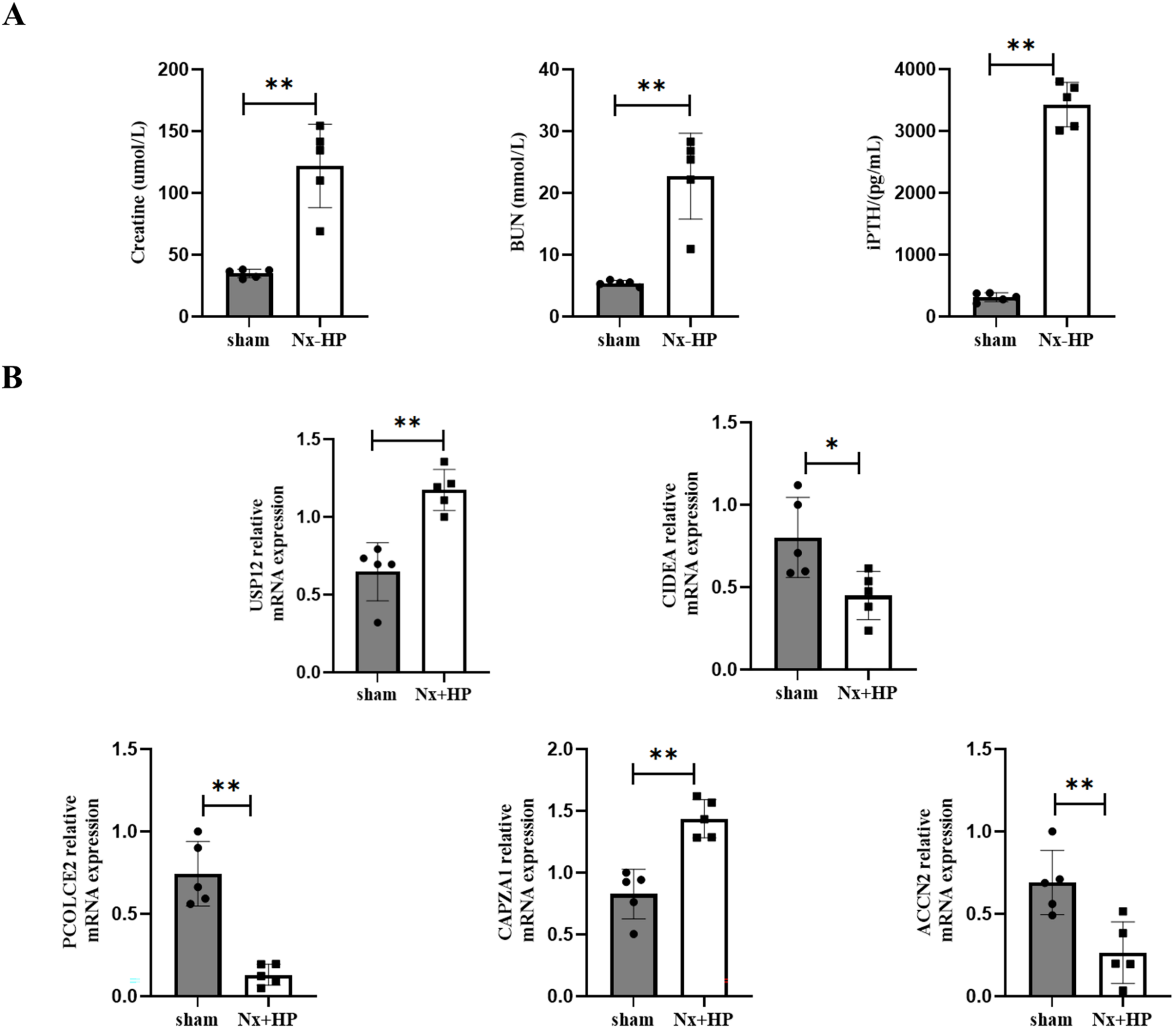
Key genes expression in animal models of SHPT. (A) The biochemistry data (serum creatinine, BUN and PTH levels) of sham-operated and SHPT rats. (B) qPCR assay to validate expression levels of key genes. *n* = 5, *p < 0.05, **p < 0.01.

A 5/6-nephrectomy (Nx) rat fed with high dietary phosphate, as was done here, is a commonly used animal model of human SHPT (Zhang et al., 2011). Serum creatinine, BUN, and PTH levels were significantly higher in the Nx+HP group compared with the sham (Fig. 8A). The mRNA expression of CAPZA1 and USP12 in the parathyroid glands of SHPT rats was elevated, while that of CIDEA, PCOLCE2, and ACCN2 was decreased compared with sham rats. The results were similar to those seen in hyperplastic parathyroid tissues of SHPT patients (Fig. 8B).

## Discussion

To the best of our knowledge, our study is the first to perform comprehensive bioinformatics analysis in SHPT. In this study, we searched NCBI-GEO databases for datasets about SHPT and conducted bioinformatics analysis to elucidate key genes which may be associated with the proliferation of the disease. Five key genes were screened from the differential genes in the GEO database by machine learning methods and verified in animal models and patient tissue samples: USP12, CIDEA, PCOLCE2, CAPZA1, and ACCN2. The molecular mechanism of these genes was further explored by predicting transcription factors and miRNAs-lncRNAs. Disease correlation analysis confirmed that these genes are related to the pathogenesis of SHPT. These findings may provide new insights into the hyperplasia mechanism of SHPT.

Under physiological conditions, parathyroid cells are generally quiescent. In advanced CKD, however, parathyroid cells gradually become active and invasive, and do not respond to conventional drug therapies (Naveh-Many & Volovelsky, 2020). Many studies have reported that the mechanisms related to parathyroid hyperplasia may also play an important role in the proliferation of tumor cells; for example, the increase of parathyroid cell cycle progression in SHPT is related to decreased p21 and p27 expression, while their changes are also related to many human malignant tumors (Graña & Reddy, 1995; Naveh-Many & Volovelsky, 2020). As such, SHPT may share similar mechanisms to tumor proliferation, possibly related to the key genes identified. USP12 is a member of the ubiquitin-specific protease family of deubiquitinases which have been identified as potential therapeutic targets for various diseases including cancers in which ubiquitin-protein homeostasis pathways are disrupted (Aron et al., 2018). For example, USP12 is involved in the development of breast cancer, lung cancer, and prostate cancer (McClurg et al., 2018; Sheng et al., 2021; Yang et al., 2021). CAPZA1 can regulate the actin cytoskeleton remodeling; dynamic reorganization of actin cytoskeleton is a prerequisite for morphogenesis and metastasis of tumor cells (Huang, Cao & Zheng, 2017). Both USP12 and CAPZA1 were found to play an important role in SHPT. The expression of CIDEA in hyperplasia was lower than that in normal parathyroid glands and adenomas (Szende et al., 2006), indicating that CIDEA may contribute to disease progression in SHPT through inhibition of apoptosis. ACCN2 contributes to the influx of Ca^2+^ in pulmonary artery smooth muscle cells (Jernigan, 2015), and small oscillations in extracellular Ca^2+^ may induce large variations in PTH production (Portillo & Rodríguez-Ortiz, 2017). This suggests that ACCN2 may affect the development of SHPT. Tonsil-derived mesenchymal stem cells can increase reactive oxygen species (ROS) production of neutrophils through PCOLCE2, thus enhancing host defense (Yoon et al., 2022). Within living cells, mitochondria are considered relevant sources of ROS (Willems et al., 2015). Our previous retrospective analysis showed that mitochondrial mass and the mitochondrial-associated protein in oxyphil cell nodules are increased compared to those in chief cell nodules. Moreover, the synthesis and secretion of PTH in oxyphil cell nodules were significantly higher than in chief cell nodules (Mao et al., 2022). Therefore, PCOLCE2 may be related to the mitochondria of the parathyroid gland in SHPT patients. Taken together, these markers may prove to be useful in predicting disease progression and prognosis, and provide novel potential targets for future SHPT research.

The influence of the tumor immune microenvironment on the survival of patients has been confirmed in a variety of different types of cancer (Li et al., 2017). Normal responses of the innate and adaptive immune systems are impaired in CKD patients (Syed-Ahmed & Narayanan, 2019). Consequently, we assessed the immune microenvironment and the proportion of immune cells in diffuse and nodular hyperplasia parathyroid glands. In the immune infiltration analysis, the level of NK cells activated was significantly lower in the nodular hyperplasia group than the diffuse hyperplasia group. The five key genes identified are closely related to immune cell infiltration levels and play important roles in the immune microenvironment; however, the specific effect on immune infiltration of parathyroid hyperplasia needs further study.

Most importantly, our approach is significantly different from previous bioinformatics reports which only identified differential genes or key genes. We took this a step further and analyzed the upstream regulation of the key genes, finding that ACCN2, USP12, and PCOLCE2 were regulated by several transcription factors. Enrichment analysis of these transcription factors found that ZBTB6 was the most significantly enriched motif for the key genes, and so may be a potential target for regulating SHPT proliferation. Previous studies have suggested that ZBTB6 may be an energy metabolism-based gene correlated with clinical outcomes in esophageal carcinomas (Zheng et al., 2021).

Small non-coding RNA is also a regulator of gene expression which can regulate hormone synthesis, hormone release, and endocrine cell proliferation. miRNAs are abundant small non-coding RNAs that can regulate hormone response and participate in the occurrence and development of endocrine tumors (Vaira et al., 2017). miRNAs are involved in regulating PTH release from normal parathyroid cells stimulated by low calcium (Shilo et al., 2015). In addition, Let-7 and microRNA-148 have been found to regulate parathyroid hormone levels in SHPT (Shilo et al., 2017). In this study, we identified miRNAs and lncRNAs of USP12, PCOLCE2, and CAPZA1 through prediction and analysis of the database. Our results suggest that Let7 also plays an important role in SHPT. Meanwhile, miRNA-3619 and SNHG14 are the highest-ranked miRNA and lncRNA, respectively, which provides a direction for the research of noncoding RNAs in SHPT. In summary, the relationship between miRNAs and SHPT is very complex, and miRNAs can participate in the occurrence and development of SHPT through various pathways. In future research, we need to delve deeper into the interaction between miRNAs and SHPT, to provide more effective measures for the clinical treatment of SHPT.

The relationship between key genes and SHPT was also analyzed using the GeneCards database to verify that the expression levels of key genes were significantly correlated with the expression levels of known parathyroid-related disease-causing genes such as VDR, FGF23, and CaSR. CAPZA1 was found to be positively correlated with CDKN1B which had decreased expression in parathyroid tumors of patients with CKD (Lauter & Arnold, 2008). Meanwhile, ACCN2, PCOLCE2, USP12, and CAPZA1 were also highly correlated to GNAS which is associated with parathyroid disease (Bastepe, 2018). Furthermore, the AUC in the ROC analyses of the five key genes were all greater than 0.8, showing relatively high specificity and sensitivity predictive performance for SHPT. We verified the expression of those key genes in various samples of the parathyroid glands from uremic patients and SHPT animal models. These results indicate that USP12, CIDEA, PCOLCE2, CAPZA1, and ACCN2 may be potential markers in parathyroid proliferation progression to SHPT.

Tycova et al. have identified different candidate genes (RANGRF, EXOC8, PIN1, CACYBP) as key drivers of parathyroid growth in SHPT with the database of GSE75886 (Ttfytfcová et al., 2016). These genes were different from the key genes we analyzed, because different analysis methods will yield different results, such as different screening conditions that will result in different DEGs. And we obtained five key genes through two machine learning methods and external dataset validation after screening for differentially expressed genes in the dataset. These five key genes are associated with immunity, and the ROC curve suggests that key genes are all energized to predict the occurrence and development of SHPT.

The study has some advantages: this is the first study to use bioinformatics to comprehensively analyze SHPT to predict biomarkers of proliferation and disease progression; more accurate machine learning methods were used to screen key genes; and we expanded on gene identification to also predict the upstream transcription factors of key genes. All of these provide more research directions for potential therapeutic targets of SHPT. However, our study also has several limitations: all data were obtained from an online database, and as a result, this study had a relatively small sample size; the small validation cohort of human and rat tissues; further research with larger sample sizes and a combination of *in vitro*/*in vivo* investigations would better determine if the identified genes can be used diagnostic indicators and/or therapeutic targets for SHPT. In addition, although we identified five key genes that differed between groups, the specific mechanism of their role in hyperparathyroidism remains unclear. The results provide a direction for future research and can be added to the relevant experiments for further exploration.

## Conclusions

USP12, CIDEA, PCOLCE2, CAPZA1, and ACCN2 have great potential as therapeutic targets and biomarkers for the diagnosis of SHPT. The present study aimed to identify early biomarkers of SHPT and the proliferative molecules and detailed molecular mechanisms that affect the progress of SHPT, which may be used to improve the survival of uremic patients. Taken together, these findings provide novel biomarkers and potential directions for future SHPT research.

## Supplemental Information

10.7717/peerj.15633/supp-1 Supplemental Information 1 Raw data of Figures 7 and 8.

10.7717/peerj.15633/supp-2 Supplemental Information 2 Supplemental tables. Table S1. Clinical and biochemical characters of the ten patients who received PTx with forearm autograft. Table S2. The human gene primer pairs and sequences for RT-PCR are listed. Table S3. The rat gene primer pairs and sequences for RT-PCR are listed.

10.7717/peerj.15633/supp-3 Supplemental Information 3 Author checklist.

## Additional Information and Declarations

### Data Availability

The following information was supplied regarding data availability: The data is available at NCBI GEO: GSE75886, GSE83421.
