# Supplementary material for: Identification of key biomarkers based on the proliferation of secondary hyperparathyroidism by bioinformatics analysis and machine learning
Source: PeerJ. 2023 Jul 10;11:e15633. doi: 10.7717/peerj.15633 (PMC10340109; doi:10.7717/peerj.15633)
Supplement: Supplemental Information 2 — Table S1. Clinical and biochemical characters of the ten patients who received PTx with forearm autograft. Table S2. The human gene primer pairs and sequences for RT-PCR are listed. Table S3. The rat gene primer pairs and sequences for RT-PCR are listed. [file peerj-11-15633-s002.docx]

Table S1. Clinical and biochemical characters of the ten patients who received PTx with forearm autograft

| Characteristic | Value |
| --- | --- |
| Age(years) | 51.10±6.92 |
| Gender (male/female) | 3/7 |
| HD vintage (years) | 10.20±4.24 |
| HD frequency (Times/week) | 3.0±0.0 |
| **Pre-PTx serumbiochemical indices** |  |
| Corrected Ca (mg/dl) | 2.57±0.27 |
| Phosphate (mg/dl) | 2.00±0.44 |
| Intact PTH (pg/ml) | 1870.19±560.69 |
| **Post-PTx serumbiochemical indices** |  |
| Corrected Ca (mg/dl) | \| 2.39±0.15 \| \| --- \| |
| Phosphate (mg/dl) | \| 1.70±0.54 \| \| --- \| |
| Intact PTH (pg/ml) | \| 19.18±24.64 \| \| --- \| |

Table S2. The human gene primer pairs and sequences for RT-PCR are listed.

| Gene | Forward primer | Reverse primer |
| --- | --- | --- |
| USP12 | 5’-AACACTTCAGGTGATGCCA-3’ | 5’-ATTGGGACCACTTCCACAG-3’ |
| CIDEA | 5’-TCAGCAAGACTCTGGATGC-3’ | 5’-ACTCTTCTGTGTCCACCAC-3’ |
| PCOLCE2 | 5’-CAAGTGACTTTGTATTAGCCGG-3’ | 5’-TGATGATCGAGACTGTGGC-3’ |
| CAPZA1 | 5’-TATTCAATGACGTTCGGCT-3’ | 5’-TGTTATACTGGGCAAATGCA-3’ |
| ACCN2 | 5’-TCCGCTTTAGCCAAGTCTC-3’ | 5’-GTATCTCATACCTGTTGTTGAGC-3’ |

Table S3. The rat gene primer pairs and sequences for RT-PCR are listed.

| Gene | Forward primer | Reverse primer |
| --- | --- | --- |
| USP12 | 5’-TAACACTTCAGGAGACGCA-3’ | 5’-ATTGGGACCACTTCCACAG-3’ |
| CIDEA | 5’-GAGTTCTTTCAGACCCTAAGAG-3’ | 5’-ATACTTATTGCCCGGTGTC-3’ |
| PCOLCE2 | 5’-CGAAGAACCAGCTCATAGAG-3’ | 5’-CAGCCACGTAGTCATATCTG-3’ |
| CAPZA1 | 5’-CATTTGCCCAGTATAACATGGA-3’ | 5’-CATGCTCTGTAATTAAGACCTGG-3’ |
| ACCN2 | 5’-AGCAGAGGCTCATCTACCT-3’ | 5’-TGTAGGAGTCGAAGAAATCCG-3’ |
